# A four-parameter laboratory risk stratification model at admission for 90-day mortality in hepatorenal syndrome-acute kidney injury: a single-center derivation and internal validation study

**DOI:** 10.3389/fneph.2026.1796660

**Published:** 2026-07-17

**Authors:** Julian Müller-Kühnle, Mathias Becker, Severin Schricker, Wolfram Zoller, Jörg Latus, Moritz Schanz

**Affiliations:** 1 Department of General Internal Medicine and Nephrology, Robert Bosch Krankenhaus (Robert Bosch Hospital), Stuttgart, Germany; 2 Paracelsus Medizinische Universität (Paracelsus Medical University), Salzburg, Austria; 3 Department of Cardiology and Angiology, Robert Bosch Krankenhaus (Robert Bosch Hospital), Stuttgart, Germany; 4 Department of General Internal Medicine, Gastroenterology, Gastrointestinal Oncology, Hepatology, Infectious Diseases, and Pulmonology, Klinikum Stuttgart (Stuttgart Hospital), Stuttgart, Germany

**Keywords:** acute kidney injury, cirrhosis, hepatorenal syndrome, inflammation, intensive care, MELD, prognostic model, risk prediction

## Abstract

**Background:**

Hepatorenal syndrome-acute kidney injury (HRS-AKI) is a high-mortality complication of advanced cirrhosis. Early risk stratification after hospital admission may support clinical assessment and research on escalation pathways, but simple admission-based models that avoid in-hospital data leakage remain limited.

**Methods:**

We performed a retrospective single-center derivation and internal-validation study of hospitalized adults with chart-review-confirmed HRS-AKI according to contemporary ADQI/ICA criteria. Candidate predictors were restricted *a priori* to admission laboratory values to minimize data leakage. Admission variables were prioritized using random-forest importance rankings under prespecified parsimony constraints, followed by endpoint-specific closed-form logistic regression models using an identical four-predictor set across outcomes. The primary endpoint was 90-day all-cause mortality. In-hospital hemodialysis and ICU admission were prespecified secondary endpoints and are reported as exploratory. Internal validation used bootstrap resampling with assessment of apparent and optimism-corrected discrimination and overall accuracy; calibration slope and calibration-in-the-large/intercept were reported descriptively. For contextual benchmarking, admission MELD, MELD-Na, and MELD 3.0 were evaluated as single-predictor logistic benchmark models for 90-day mortality within the same cohort.

**Results:**

The final analysis included 77 patients. The final predictor set comprised INR, albumin, ln(total bilirubin), and ln(C-reactive protein). For 90-day mortality, the final four-laboratory model showed an apparent AUC of 0.781 (95% CI, 0.715-0.926) and an optimism-corrected AUC of 0.758 (95% CI, 0.648-0.859), with an apparent Brier score of 0.144 (95% CI, 0.081-0.176), an optimism-corrected Brier score of 0.168 (95% CI, 0.117-0.212), an optimism-corrected calibration slope of 0.770, and an optimism-corrected calibration-in-the-large/intercept of 0.168. Performance for in-hospital hemodialysis and ICU admission was limited, with optimism-corrected AUCs of 0.580 (95% CI, 0.451-0.733) and 0.631 (95% CI, 0.514-0.737), respectively. MELD-family benchmarks showed numerically lower within-cohort discrimination than the four-laboratory model; these comparisons were descriptive and hypothesis-generating.

**Conclusions:**

In this single-center derivation and internal-validation study, an admission-based four-laboratory model showed moderate internally validated discrimination for 90-day mortality in hospitalized patients with HRS-AKI. The model should be interpreted as a preliminary risk-stratification approach requiring external validation and, if necessary, recalibration before clinical use. Secondary endpoint models for hemodialysis and ICU admission are exploratory and should not be used for threshold-based decision-making.

## Introduction

Hepatorenal syndrome-acute kidney injury (HRS-AKI) is a life-threatening complication of advanced cirrhosis characterized by severe circulatory dysfunction and rapidly progressive kidney impairment, with high short-term morbidity and mortality (1–4). In the acute care setting, clinicians must often make time-sensitive decisions soon after admission - such as intensified monitoring, early nephrology involvement, dialysis preparedness, and transplant evaluation - under substantial prognostic uncertainty (3, 4).

Although commonly used cirrhosis severity scores capture important elements of systemic illness, they were not developed specifically for HRS-AKI admission risk stratification and may not provide simple admission-based probability estimates from a fixed minimal laboratory set (5–7). Moreover, prognostic modeling in hospitalized cohorts is vulnerable to data leakage, where predictors inadvertently encode information from the subsequent hospital course, yielding misleadingly optimistic performance (8). This risk is particularly salient in small cohorts, where split-sample approaches are unstable and rigorous resampling-based internal validation is preferred (9, 10). Transparent reporting in line with TRIPOD guidance is therefore essential (11).

We aimed to develop and internally validate a parsimonious admission-based risk-stratification model for 90-day mortality in hospitalized patients with HRS-AKI using routinely available admission laboratory values. Predictors were restricted *a priori* to the admission window to minimize data leakage. Machine learning–guided variable prioritization was combined with closed-form logistic regression to preserve transparency and interpretability. Prespecified secondary objectives were exploratory models for in-hospital hemodialysis and ICU admission, given their dependence on practice patterns, competing risks, and limited event counts.

## Materials and methods

### Study design and setting

We conducted a single-center retrospective cohort study at Robert Bosch Hospital (Robert Bosch Krankenhaus), Stuttgart, Germany. The study period extended from January 2019 to September 2024. Consecutive adult patients with ICD-10-coded hepatorenal syndrome were screened for eligibility.

### Study population and case ascertainment

Eligible patients were adults (≥18 years) with cirrhosis and HRS-AKI according to contemporary ADQI/ICA consensus criteria (4). Patients were initially identified through ICD-10 coding for hepatorenal syndrome and subsequently underwent structured chart review by two independent reviewers from the nephrology department. Disagreements or uncertain cases were resolved by nephrology consensus review.

For case ascertainment, baseline creatinine was defined as the first available serum creatinine measurement at hospital admission. HRS-AKI confirmation required chart-review evidence of cirrhosis with ascites and AKI, defined as an increase in serum creatinine of ≥0.3 mg/dL within 48 hours or ≥1.5-fold from baseline within 7 days (4). Cases were excluded if shock, sepsis at admission, relevant nephrotoxic drug exposure, obstructive uropathy, structural kidney disease, or another identifiable cause was considered the primary explanation for kidney dysfunction. Patients whose kidney function improved after albumin challenge were not classified as HRS-AKI and were excluded. Case ascertainment also incorporated documented volume status, diuretic withdrawal, clinically indicated volume expansion, and vasoconstrictor treatment.

The admission-based design was chosen to preserve temporal clarity and minimize data leakage. HRS-AKI status was confirmed by structured chart review, whereas predictor eligibility was restricted to the first available hospital-admission laboratory values, as detailed below. Thus, the model provides early prognostic risk stratification in patients with confirmed HRS-AKI using information available from the admission window.

Urinary findings and imaging reports were reviewed when available as part of routine clinical evaluation to support case ascertainment and exclude alternative diagnoses. These included urine studies documented in the medical record and imaging reports such as abdominal ultrasound, radiography, computed tomography, or magnetic resonance imaging, depending on clinical availability. These data supported adjudication only and were not included as candidate predictors. Cases with insufficient documentation to confirm HRS-AKI were excluded from the final analytic cohort.

### Outcomes

We evaluated three outcomes: 90-day all-cause mortality after hospital admission as the primary outcome, and in-hospital hemodialysis and ICU admission during the index hospitalization as exploratory secondary outcomes.

Ninety-day all-cause mortality was ascertained retrospectively from existing institutional medical records, including deaths documented during the index hospitalization and post-discharge survival information documented during subsequent hospital encounters or communicated by treating physicians as part of routine clinical care. Ninety-day survival status was available for all patients in the final analytic cohort.

Hemodialysis and ICU admission were recorded as binary in-hospital outcomes. ICU admission was defined as any ICU admission during the index hospitalization, including direct ICU admission or later transfer. Patients who died before dialysis initiation were classified as “no dialysis” according to the recorded endpoint definition, introducing a competing-risk limitation for the hemodialysis endpoint. Because hemodialysis initiation and ICU admission may be influenced by transplant candidacy, treatment goals, clinical decision-making, resource availability, and center-specific practice patterns, both secondary outcome models were considered exploratory.

### Data processing and missing data

We applied a prespecified preprocessing workflow with standardized units and coding. Clinically implausible values consistent with data entry errors were set to missing after plausibility checks. Missing predictor values were handled using prespecified single imputation, with the median used for continuous variables and the mode used for binary variables. Outcomes were not imputed. Total bilirubin and CRP were natural log-transformed to reduce right-skew and the influence of extreme values. Missingness was assessed for all candidate predictors considered during model development. The missingness pattern and the imputation values used for final model calculation are reported in **Supplementary Table 1**.

### Model development

Candidate predictors were restricted *a priori* to the first available laboratory values from the hospital-admission window and were defined before any endpoint occurrence. Variables reflecting later diagnostic evolution, treatment response, or the subsequent in-hospital course were not considered.

Candidate variables were prioritized using random-forest classifiers trained separately for each outcome, with 600 trees, a fixed random seed, and class-weighted fitting to address class imbalance. Random forests were used only as an exploratory tool for predictor prioritization among admission candidate variables and were not used as final prediction models.

Final logistic regression models were prespecified to use a parsimonious admission-laboratory predictor set to preserve interpretability, feasibility, and leakage-resistant implementation. Selection of the final common four-variable set was based on random-forest rankings, clinical plausibility, routine availability, non-redundant representation of key disease domains, and feasibility of transparent closed-form calculation. Random-forest variable-importance plots are provided in **Supplementary Figure 1**.

Final models were endpoint-specific logistic regressions specified in closed form to ensure transparent calculation and reproducibility; coefficients and implementation details are reported in **Table 1**.

**Table 1:** Final closed-form logistic regression models.

Outcome	Intercept (β0)	INR (β1)	Albumin, g/dL (β2)	ln (total bilirubin, mg/dL) (β3)	ln (CRP, mg/dL) (β4)
90-day mortality	-0.2001966	1.7074356	-0.7025705	0.0920138	0.60496
In-hospital hemodialysis	-0.9948261	0.0074999	-0.0847456	0.2289445	-0.47056
ICU admission	3.1704776	0.4763276	-0.6330387	0.0156324	-0.58696

Models were fitted using endpoint-specific unpenalized logistic regression with an identical four-variable admission-laboratory predictor set for all outcomes. For each outcome, the linear predictor is calculated as LP = β0 + β1·INR + β2·Albumin + β3·ln(Total bilirubin) + β4·ln(CRP), and the predicted probability is P = 1/(1+exp(−LP)). ln denotes the natural logarithm. Units are as shown in the table; INR is unitless, albumin is reported in g/dL, total bilirubin in mg/dL, and CRP in mg/dL. Missing predictor values were handled using prespecified single imputation before calculation of LP and P. For the final four-variable model, imputation values were INR 1.54, albumin 2.67 g/dL, and total bilirubin 4.5 mg/dL; CRP had no missing values. Complete missingness and imputation details for all candidate predictors are provided in **Supplementary Table 1**. CRP, C-reactive protein; ICU, intensive care unit; INR, international normalized ratio.

### Model equation and probability calculation

For each endpoint, the linear predictor was calculated as:

LP = β0 + β1·INR + β2·Albumin + β3·ln(Total bilirubin) + β4·ln(CRP),

followed by transformation to the predicted probability:

P = 1/(1+exp(-LP)).

All predictors used the units shown in **Table 1**: INR is unitless, albumin is reported in g/dL, total bilirubin in mg/dL, and CRP in mg/dL. If a required predictor value was missing, it was first replaced by the prespecified single-imputation value and then used for LP and probability calculation, as detailed in **Table 1** and **Supplementary Table 1**.

### Internal validation and performance assessment

Internal validation used bootstrap resampling with 1,000 resamples. Apparent performance was calculated in the original cohort. Optimism correction was performed by refitting the final fixed four-variable logistic regression model in each bootstrap sample, evaluating performance in both the bootstrap sample and the original cohort, and subtracting mean optimism from the apparent estimate.

Discrimination was quantified using the AUC, overall accuracy using the Brier score, and calibration using calibration slope and calibration-in-the-large/intercept. Apparent and optimism-corrected estimates are reported for AUC and Brier score, with bootstrap 95% intervals calculated from the empirical distribution of the bootstrap resamples. Calibration slope and calibration-in-the-large/intercept are reported as descriptive optimism-corrected point estimates because interval estimation for these calibration metrics was unstable and non-informative in this small cohort with endpoint imbalance.

**Figure 1:**
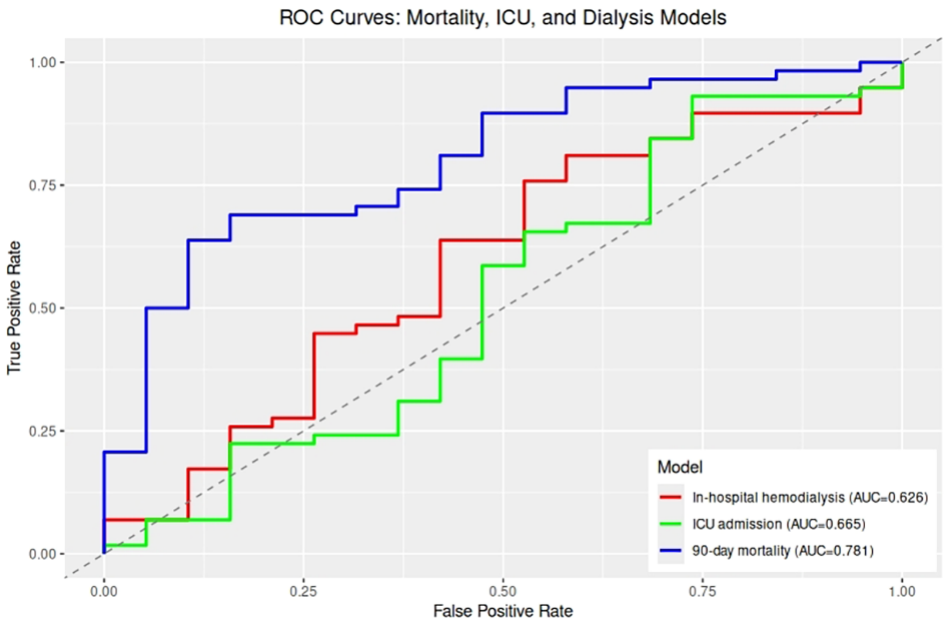
Receiver operating characteristic (ROC) curves for admission-based models. Receiver operating characteristic (ROC) curves showing discrimination of the admission-laboratory logistic regression models for 90-day all-cause mortality, in-hospital hemodialysis, and ICU admission in the derivation cohort (n=77 for all endpoints). Predicted probabilities were computed from closed-form equations using INR, albumin, ln(total bilirubin), and ln(C-reactive protein). AUCs were 0.781 for 90-day mortality, 0.626 for in-hospital hemodialysis, and 0.665 for ICU admission. The hemodialysis and ICU models are exploratory and should be interpreted cautiously given limited hemodialysis event counts, competing-risk considerations, and ICU class imbalance. ln denotes the natural logarithm.

**Figure 2:**
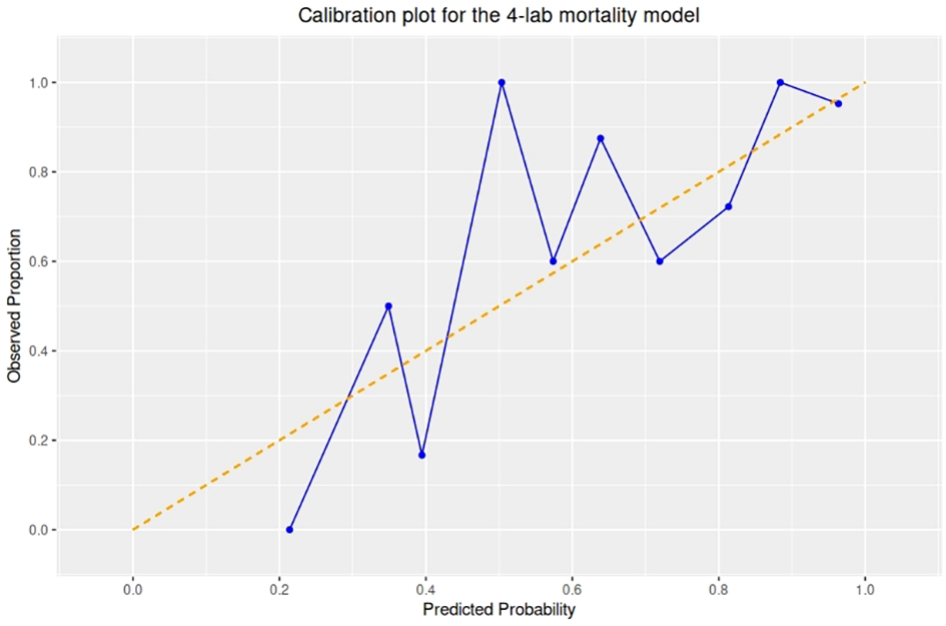
Descriptive calibration plot for the 90-day mortality model. Descriptive calibration plot for the admission-based four-laboratory model for 90-day all-cause mortality in the derivation cohort (n=77). Patients were grouped according to predicted risk for graphical display; points represent the mean predicted probability within each group and the corresponding observed event proportion. The dashed diagonal line indicates perfect calibration. Given the small cohort size and high event rate, grouped calibration is intended for descriptive visualization only and should not be interpreted as formal evidence of calibration. Predicted probabilities were generated from the closed-form logistic regression model using INR, albumin, ln(total bilirubin), and ln(C-reactive protein), with prespecified median imputation for missing predictor values.

ROC curves are shown in **Figure 1**. Calibration plots are shown for the primary endpoint in **Figure 2** and for exploratory secondary endpoints in **Supplementary Figure 2**. These plots were generated by grouping patients according to predicted risk and are intended for descriptive visualization only, not formal inference. For the mortality model, two illustrative operating points derived from ROC characteristics are reported for derivation-cohort interpretation only and require external validation and potential recalibration before clinical use.

### Contextual benchmarking against MELD-family scores

**Figure 3:**
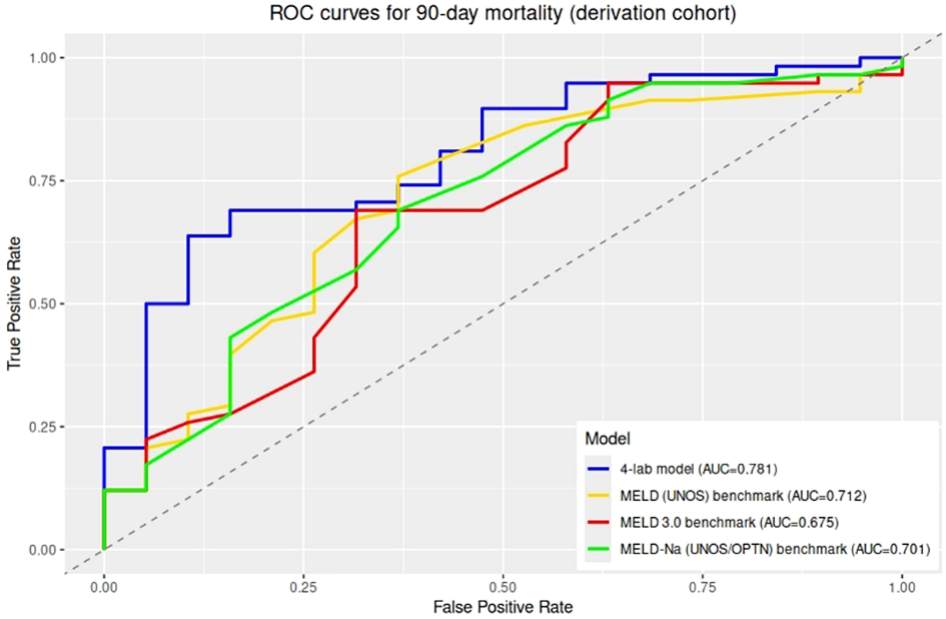
ROC curves for 90-day mortality comparing the four-laboratory model versus MELD, MELD-Na, and MELD 3.0. Receiver operating characteristic (ROC) curves showing apparent discrimination for 90-day all-cause mortality in the derivation cohort (n=77). The four-laboratory admission model provides predicted probabilities from the final closed-form equation using INR, albumin, ln(total bilirubin), and ln(CRP). MELD, MELD-Na, and MELD 3.0 were calculated from admission laboratory values and evaluated as single-predictor logistic benchmark models. Apparent AUCs were 0.781 for the four-laboratory model, 0.712 for MELD, 0.701 for MELD-Na, and 0.675 for MELD 3.0. The dashed diagonal indicates chance performance. This comparison is descriptive and intended for within-cohort benchmarking only, not to establish generalizable superiority. AUC, area under the receiver operating characteristic curve; CRP, C-reactive protein; INR, international normalized ratio; MELD, Model for End-Stage Liver Disease.

To address a clinically relevant comparator question while minimizing comparator-selection bias, we calculated admission MELD, MELD-Na, and MELD 3.0 and evaluated each as a single-predictor logistic benchmark model for 90-day all-cause mortality using the same bootstrap internal-validation framework with 1,000 resamples. MELD was calculated using the standard formulation (6), with bilirubin, INR, and creatinine values <1.0 set to 1.0 and creatinine capped at 4.0 mg/dL. Per OPTN/UNOS specifications, creatinine was set to 4.0 mg/dL for MELD and MELD-Na if the patient had received ≥2 dialysis treatments within the prior week; no patient in this cohort met this criterion. MELD-Na was calculated with serum sodium bounded between 125 and 137 mmol/L (12). MELD 3.0 was calculated according to the original published specification, with standard rounding to the nearest integer (13). A combined ROC comparison for the four-laboratory model and the MELD-family benchmarks is presented in **Figure 3**.

### Competing-risk sensitivity analysis for hemodialysis

Given the limited number of hemodialysis events and the fact that death before dialysis initiation precludes subsequent dialysis, we performed a *post hoc* Fine-Gray competing-risk sensitivity analysis for the hemodialysis endpoint. Dialysis initiation was defined as the event of interest, death before dialysis as the competing event, and patients without either event were censored. The same four predictors used in the logistic hemodialysis model were entered into the competing-risk model. This analysis was exploratory and was not used for model selection, threshold derivation, or clinical decision-making.

### Statistical analysis and software

Continuous variables are summarized as median [interquartile range], and categorical variables as n (%). Analyses were performed in Python 3.13.5 (Python Software Foundation, Wilmington, DE, USA). Data processing and numerical operations used pandas 2.2.3 and NumPy 2.3.5. Random-forest classifiers, logistic regression models, receiver operating characteristic analyses, bootstrap resampling, and performance metrics were implemented using scikit-learn 1.8.0. Figures were generated using Matplotlib 3.10.8.

Results were independently checked using RStudio 2026.5.0.218 on R version 4.6.0. The following R packages were used for reproducibility checks and supplementary analyses: cmprsk_2.2–12 for competing-risk analyses, caret_7.0–1 for performance metrics including Brier scores, pROC_1.19.0.1 for ROC curves and AUC estimation, boot_1.3–32 for bootstrap validation, purrr_1.2.2 for functional programming, tidyr_1.3.2 for data preparation, dplyr_1.2.1 for data processing, readxl_1.5.0 for reading the data file, and performance_0.16.0 and car_3.1–3 for linearity and collinearity assessment. For random number-based operations, the seed was initialized as 42.

Reporting was structured to align with TRIPOD recommendations for prediction-model development and internal validation studies (11). We did not perform a formal PROBAST risk-of-bias assessment; PROBAST is referenced in the Discussion as a relevant framework for appraisal in future external validations (14).

## Results

### Cohort and endpoint availability

Between January 2019 and September 2024, 174 ICD-10-coded hepatorenal syndrome cases were identified. After removal of duplicate or repeated episodes (n=30) and records without HRS (n=2), 142 unique patient records underwent detailed chart review. Patients were excluded from the HRS-AKI cohort because they had no AKI within 7 days after admission (n=48), no confirmed cirrhosis with ascites (n=16), or structural kidney disease as the primary explanation for kidney dysfunction (n=1). The final analytic cohort comprised 77 patients with chart-review-confirmed HRS-AKI according to contemporary ADQI/ICA criteria. Patient flow and exclusion reasons are summarized in **Supplementary Table 2**.

Baseline characteristics and admission laboratory values are summarized in **Table 2**. Outcome frequencies were 90-day mortality in 58/77 patients (75.3%), in-hospital hemodialysis in 17/77 patients (22.1%), and ICU admission in 63/77 patients (81.8%). Terlipressin exposure during hospitalization was documented in 58/77 patients as part of routine clinical care. No patient underwent liver transplantation during the 90-day observation period. Treatment variables were not included as predictors because the model was restricted *a priori* to admission laboratory values.

**Table 2:** Baseline cohort characteristics at hospital admission.

Variable	Total cohort (n=77)
Demographics	
Age, years	63 [53-73]
Male sex, n (%)	50 (64.9)
Cirrhosis etiology, n (%)	
Alcohol-related*	56 (72.7)
Viral hepatitis (HBV/HCV)†	4 (5.2)
NASH	2 (2.6)
Cryptogenic	3 (3.9)
Autoimmune hepatitis	1 (1.3)
Wilson disease	1 (1.3)
Other specified causes‡	3 (3.9)
Unknown	7 (9.1)
Liver disease severity	
Child-Pugh class (A/B/C), n (%)	A: 1 (1.3); B: 19 (24.7); C: 57 (74.0)
Comorbidities/clinical features, n (%)	
Arterial hypertension	26 (33.8)
Diabetes mellitus	19 (24.7)
Hepatic encephalopathy	38 (49.4)
Smoking	18 (23.4)
Admission vital signs	
Systolic blood pressure, mmHg	117 [100-137]
Heart rate,/min	88 [74-107]
Admission laboratory values	
Creatinine, mg/dL	1.8 [1.3-2.6]
INR	1.54 [1.30-1.92]
Albumin, g/dL	2.7 [2.4-3.1]
Total bilirubin, mg/dL	4.5 [1.8-9.9]
C-reactive protein, mg/dL	3.2 [1.2-7.0]
Sodium, mmol/L	131 [127-137]
Outcomes	
90-day all-cause mortality, n (%)	58 (75.3)
In-hospital hemodialysis, n (%)	17 (22.1)
ICU admission, n (%)	63 (81.8)

Values are median [IQR] for continuous variables and n (%) for categorical variables, unless otherwise indicated. Baseline variables reflect admission or first available hospital-admission measurements. Missing data were as follows: systolic blood pressure, n=8; heart rate, n=9; INR, n=3; total bilirubin, n=2; and albumin, n=1. All other variables listed in the table had no missing values. HBV, hepatitis B virus; HCV, hepatitis C virus; HRS-AKI, hepatorenal syndrome-acute kidney injury; ICU, intensive care unit; INR, international normalized ratio; NASH, nonalcoholic steatohepatitis. *Alcohol-related etiology includes one mixed alcohol/HCV attribution and one chart-documented “nutritional-toxic” attribution. †Viral hepatitis refers to HBV and/or HCV as the primary etiology, exclusive of the mixed alcohol/HCV case counted under alcohol-related etiology. ‡Other specified causes include portal vein thrombosis–associated etiology, suspected drug-induced etiology related to letrozole, and multifactorial attribution.

### Predictor prioritization and final model specification

Random-forest variable-importance rankings highlighted several potentially informative admission variables across endpoints, including markers of hepatic injury, systemic inflammation, hematologic status, and liver dysfunction. Because the aim was to derive a transparent and parsimonious admission-laboratory model rather than a high-dimensional machine-learning classifier, final predictor selection additionally prioritized routine availability, clinical plausibility, interpretability, and non-redundant coverage of key pathophysiologic domains. The final common predictor set comprised four admission laboratories: INR, albumin, ln(total bilirubin), and ln(CRP), reflecting hepatic synthetic dysfunction, cholestatic/hepatocellular disease burden, and systemic inflammation. Final coefficients and the closed-form implementation for all endpoints are reported in **Table 1**.

### Internal validation performance

Bootstrap internal validation with 1,000 resamples showed the most favorable performance for the primary 90-day mortality model, although all estimates were imprecise given the small cohort size. For 90-day mortality, the apparent AUC was 0.781 (95% CI, 0.715-0.926) and the optimism-corrected AUC was 0.758 (95% CI, 0.648-0.859). The apparent Brier score was 0.144 (95% CI, 0.081-0.176), and the optimism-corrected Brier score was 0.168 (95% CI, 0.117-0.212). The optimism-corrected calibration slope was 0.770, and the optimism-corrected calibration-in-the-large/intercept was 0.168.

Performance for the exploratory secondary endpoints was limited. For in-hospital hemodialysis, the apparent and optimism-corrected AUCs were 0.626 (95% CI, 0.570-0.851) and 0.580 (95% CI, 0.451-0.733), respectively. The apparent and optimism-corrected Brier scores were 0.158 (95% CI, 0.086-0.201) and 0.181 (95% CI, 0.120-0.235), respectively. The optimism-corrected calibration slope was 0.553, and the optimism-corrected calibration-in-the-large/intercept was -0.543.

For ICU admission, the apparent and optimism-corrected AUCs were 0.665 (95% CI, 0.619-0.842) and 0.631 (95% CI, 0.514-0.737), respectively. The apparent and optimism-corrected Brier scores were 0.143 (95% CI, 0.081-0.177) and 0.162 (95% CI, 0.110-0.205), respectively. The optimism-corrected calibration slope was 0.674, and the optimism-corrected calibration-in-the-large/intercept was 0.424. Full apparent and optimism-corrected performance estimates are summarized in **Table 3**. Grouped calibration plots are presented as descriptive visualizations only because of the small cohort size, high mortality rate, limited hemodialysis event count, competing-risk considerations for hemodialysis, and ICU class imbalance.

**Table 3:** Internal validation performance and illustrative probability thresholds.

A. Internal validation (bootstrap; 1,000 resamples).								
Outcome	n	Events, n (%)	AUC apparent (95% CI)	AUC optimism-corrected (95% CI)	Brier score apparent (95% CI)	Brier score optimism-corrected (95% CI)	Calibration slope optimism-corrected	Calibration-in-the-large/intercept optimism-corrected
90-day mortality	77	58 (75.3)	0.781 (0.715-0.926)	0.758 (0.648-0.859)	0.144 (0.081-0.176)	0.168 (0.117-0.212)	0.770	0.168
In-hospital hemodialysis	77	17 (22.1)	0.626 (0.570-0.851)	0.580 (0.451-0.733)	0.158 (0.086-0.201)	0.181 (0.120-0.235)	0.553	-0.543
ICU admission	77	63 (81.8)	0.665 (0.619-0.842)	0.631 (0.514-0.737)	0.143 (0.081-0.177)	0.162 (0.110-0.205)	0.674	0.424
B. Illustrative operating points for 90-day mortality (derivation cohort only).								
Outcome	n	Threshold (P)	Intended use	Sens	Spec	PPV	NPV	Above threshold, n (%)
90-day mortality	77	≥ 0.595	Sensitivity-oriented	0.897	0.526	0.852	0.625	61 (79.2)
90-day mortality	77	≥ 0.822	Rule-in	0.638	0.895	0.949	0.447	39 (50.6)

Internal validation was performed using 1,000 bootstrap resamples. Apparent performance was calculated in the original cohort. Optimism-corrected estimates were obtained by subtracting mean bootstrap optimism from apparent performance. Bootstrap 95% intervals are reported for AUC and Brier score. Calibration slope and calibration-in-the-large/intercept are reported as descriptive optimism-corrected point estimates because interval estimation for calibration metrics was unstable and non-informative in this small cohort with endpoint imbalance. Discrimination was quantified using the area under the receiver operating characteristic curve (AUC), overall accuracy using the Brier score, and calibration using calibration slope and calibration-in-the-large/intercept. Hemodialysis and ICU admission were exploratory endpoints and should be interpreted cautiously because of limited event counts, competing-risk considerations, and class imbalance. Abbreviations: AUC, area under the receiver operating characteristic curve; CI, confidence interval; ICU, intensive care unit. Operating points for the 90-day mortality model are illustrative and were derived from ROC characteristics in the derivation cohort only. These thresholds are not recommended for clinical decision-making without external validation and, if necessary, recalibration. NPV, negative predictive value; PPV, positive predictive value; Sens, sensitivity; Spec, specificity.

### Competing-risk sensitivity assessment for hemodialysis

The hemodialysis endpoint was based on 17 events, corresponding to approximately 4.25 events per predictor in the four-variable model. This low event count supports classifying the hemodialysis equation as exploratory. In addition, the endpoint is susceptible to competing-risk bias because death before dialysis initiation was classified as “no dialysis” according to the recorded endpoint definition. A *post hoc* Fine-Gray sensitivity analysis accounting for death before dialysis as a competing event did not identify robust predictor associations. These findings support interpreting the hemodialysis model as exploratory and not suitable for threshold-based clinical decision-making.

### Contextual benchmark comparison with MELD-family scores

In this cohort, MELD-family benchmarks showed numerically lower within-cohort discrimination than the four-laboratory mortality model; however, these comparisons were descriptive and not intended to establish generalizable superiority. Apparent AUCs were 0.781 (95% CI, 0.715-0.926) for the four-laboratory model, 0.712 (95% CI, 0.571-0.846) for MELD, 0.701 (95% CI, 0.553-0.840) for MELD-Na, and 0.675 (95% CI, 0.510-0.811) for MELD 3.0. Optimism-corrected AUCs were 0.758 (95% CI, 0.648-0.859) for the four-laboratory model, 0.712 (95% CI, 0.576-0.854) for MELD, 0.697 (95% CI, 0.551-0.841) for MELD-Na, and 0.656 (95% CI, 0.496-0.812) for MELD 3.0.

Apparent and optimism-corrected Brier scores are reported in **Table 4**, together with descriptive optimism-corrected calibration slope and calibration-in-the-large/intercept estimates. Apparent ROC curves for all four approaches are shown in **Figure 3**. Given the small cohort size and high event rate, all benchmark comparisons should be interpreted as descriptive and hypothesis-generating.

**Table 4:** Benchmark comparison for 90-day mortality (MELD, MELD-Na, MELD 3.0 vs 4-lab).

Model	n	Deaths, n (%)	AUC apparent (95% CI)	AUC optimism-corrected (95% CI)	Brier score apparent (95% CI)	Brier score optimism-corrected (95% CI)	Calibration slope optimism-corrected	Calibration-in-the-large/intercept optimism-corrected
Four-laboratory admission model (fixed closed-form)	77	58 (75.3)	0.781 (0.715-0.926)	0.758 (0.648-0.859)	0.144 (0.081-0.176)	0.168 (0.117-0.212)	0.770	0.168
MELD benchmark	77	58 (75.3)	0.712 (0.571-0.846)	0.712 (0.576-0.854)	0.169 (0.112-0.209)	0.173 (0.138-0.237)	0.626	0.579
MELD-Na benchmark	77	58 (75.3)	0.701 (0.553-0.840)	0.697 (0.551-0.841)	0.166 (0.108-0.206)	0.170 (0.134-0.238)	1.127	-0.180
MELD 3.0 benchmark	77	58 (75.3)	0.675 (0.510-0.811)	0.656 (0.496-0.812)	0.168 (0.111-0.209)	0.173 (0.139-0.240)	0.875	0.129

Admission MELD, MELD-Na, and MELD 3.0 were calculated from admission laboratory values and evaluated as single-predictor logistic regression benchmark models for 90-day all-cause mortality in the derivation cohort (n=77). The four-laboratory admission model row reports the final 90-day mortality model performance for direct contextual comparison with the MELD-family benchmark models. Performance metrics are reported as apparent and optimism-corrected estimates from bootstrap internal validation with 1,000 resamples. Bootstrap 95% intervals are reported for AUC and Brier score. Calibration slope and calibration-in-the-large/intercept are reported as descriptive optimism-corrected point estimates because interval estimation for calibration metrics was unstable and non-informative in this small cohort with a high event rate and limited numbers of non-events. No patient had hemodialysis in the week prior to admission; therefore, no dialysis override was applied during MELD or MELD-Na calculation. The comparison is descriptive and not intended to establish generalizable superiority. Calibration estimates should be interpreted cautiously given the small cohort size and high event rate. AUC, area under the receiver operating characteristic curve; CI, confidence interval; MELD, Model for End-Stage Liver Disease.

## Discussion

### Principal findings and intended use case

In this retrospective single-center derivation and internal-validation study, we developed an admission-only four-laboratory risk-stratification model for 90-day mortality in hospitalized patients with HRS-AKI. The model was intentionally restricted to routinely available admission laboratory values to minimize data leakage and preserve interpretability. Its intended use at this stage is not direct clinical deployment, but preliminary risk stratification and hypothesis generation in a high-risk population in which early prognostic assessment is clinically relevant.

This design targets a narrow but important prognostic window: early risk stratification using laboratory values available from hospital admission among patients with confirmed HRS-AKI. Because HRS-AKI confirmation may require short-term clinical evolution and response assessment, the model should not be interpreted as a diagnostic tool available at the moment of admission. Rather, it is intended to estimate prognosis in patients whose HRS-AKI status has been confirmed by structured chart review while preserving an admission-window predictor definition.

### Performance of the primary mortality model and interpretation

For the primary endpoint, bootstrap internal validation demonstrated moderate but imprecisely estimated discrimination for 90-day mortality. The four-laboratory mortality model had an apparent AUC of 0.781 and an optimism-corrected AUC of 0.758, with an apparent Brier score of 0.144 and an optimism-corrected Brier score of 0.168. Bootstrap 95% intervals for AUC and Brier score are reported in the Results and in **Table 3**. The optimism-corrected calibration slope was 0.770, and the optimism-corrected calibration-in-the-large/intercept was 0.168. These calibration metrics are reported as descriptive point estimates because interval estimation for calibration was unstable and non-informative in this small cohort with a high event rate and only 19 non-events.

Overall, these estimates indicate residual optimism and substantial uncertainty, which are expected in small derivation cohorts and reinforce the need for external validation and likely recalibration before any threshold-based clinical application. To avoid overstating readiness for threshold-based decision-making, we present probability cut points only as illustrative operating points in the derivation cohort, not as deployable clinical cutoffs. This conservative framing is aligned with established recommendations for transparent reporting and cautious interpretation of internally validated prognostic models (11) and with structured appraisal principles emphasized in risk-of-bias tools for prediction modeling (14).

### Benchmarking against MELD, MELD-Na, and MELD 3.0

An important comparative question is whether an admission-based model provides prognostic information beyond MELD-family scores. We addressed this directly while minimizing comparator-selection bias by benchmarking against MELD, MELD-Na, and MELD 3.0, each evaluated within the same cohort under the same bootstrap framework. This all-three strategy reflects heterogeneous real-world practice and reduces dependence on any single MELD-family comparator (6, 12, 13).

In this cohort, MELD-family benchmarks showed numerically lower within-cohort discrimination than the four-laboratory model, both before and after optimism correction. These results are best interpreted as contextual evidence that an admission-based model incorporating inflammation and hepatic synthetic function may capture prognostic signal that is not fully represented by MELD-family constructs in this hospitalized HRS-AKI setting. They are not, by design, definitive evidence of superiority across centers: MELD-family performance can vary by case mix, assay practices, and operational definitions, and transportability of both discrimination and, critically, absolute-risk calibration must be established through external validation.

### Biological coherence and clinical plausibility of predictors

The final predictor set maps to clinically coherent domains in advanced cirrhosis and HRS-AKI. INR and albumin reflect hepatic synthetic dysfunction, total bilirubin captures cholestatic/hepatocellular dysfunction and overall liver disease burden, and CRP provides a pragmatic marker of systemic inflammation and infection, which are frequent precipitants of acute decompensation. Prior work supports CRP as an independent short-term mortality marker in cirrhosis (15). More broadly, systemic inflammation is increasingly recognized as a major driver of organ failure and short-term outcomes in acute decompensation and related syndromes, supporting the biological plausibility of inflammation-sensitive admission risk stratification (16, 17).

Random-forest rankings were used as one component of a broader variable-prioritization process and should not be interpreted as a mandate to select the four highest-ranked variables. Final predictor selection also considered parsimony, routine availability, interpretability, non-redundancy, and suitability for closed-form logistic regression. Because random-forest prioritization and final predictor selection were performed in the same small single-center cohort, bootstrap validation of the fixed final model may not fully capture uncertainty introduced by the preceding variable-selection step. Therefore, the apparent numerical advantage over MELD-family scores should be interpreted strictly as descriptive and hypothesis-generating. Variables with high random-forest importance, such as AST, hemoglobin, leukocytes, and lactate, may reflect acute hepatocellular injury, hematologic status, infection, bleeding, tissue hypoperfusion, or general illness severity; however, they may also be more context-dependent and less stable in a small derivation cohort.

### Secondary endpoints: why they are exploratory in this derivation study

Secondary endpoints, namely in-hospital hemodialysis and ICU admission, showed limited predictive performance compared with the primary mortality model. This limited performance was clinically plausible given the endpoint structure, low event count for hemodialysis, and class imbalance for ICU admission. The negative ln(CRP) coefficients in the exploratory hemodialysis and ICU models should not be interpreted as evidence of a protective inflammatory effect. They more likely reflect model instability, endpoint structure, competing-risk mechanisms, class imbalance, and residual confounding in a small derivation cohort.

The hemodialysis model was based on only 17 events and is susceptible to competing-risk bias because patients who died before dialysis initiation were classified as “no dialysis” according to the recorded endpoint definition. Hemodialysis initiation may reflect not only physiological severity but also transplant candidacy, anticipated reversibility, clinician decision-making, treatment goals, and center-specific practice patterns. A *post hoc* Fine-Gray sensitivity analysis accounting for death before dialysis as a competing event did not identify robust predictor associations. These findings reinforce that the hemodialysis equation should not be used for threshold-based clinical decisions and that future dialysis prediction in HRS-AKI would require larger cohorts, time-to-event information, competing-risk modeling, and careful accounting for treatment goals and transplant candidacy.

ICU admission was also imbalanced, with 63 ICU admissions among 77 patients, limiting both discrimination and calibration assessment. In addition, ICU admission may partly reflect admission pathways and local resource utilization rather than a purely future clinical event. These considerations reinforce our prespecified interpretation of the secondary equations as exploratory, hypothesis-generating analyses that are not suitable for threshold-based clinical decisions without external validation and endpoint-specific modeling strategies. This stance is consistent with contemporary recommendations that emphasize sample-size adequacy and optimism control during prognostic model development (18).

### Strengths

Several features strengthen the interpretability and relevance of this work. First, the strict admission-only predictor window minimized data leakage, a common but often underappreciated source of inflated model performance in hospital-based prediction research (8). Second, the final model deliberately prioritizes parsimony and transparency by using four routinely available laboratory tests in a closed-form logistic equation. Third, HRS-AKI case ascertainment was based on structured chart review by two independent nephrology reviewers rather than ICD-10 coding alone. Fourth, bootstrap internal validation with optimism correction provided more appropriate performance estimates than split-sample methods in this small cohort (9, 11). Finally, mortality probability thresholds are reported only as derivation-cohort operating points rather than recommended clinical cutoffs.

### Limitations

Several limitations should be considered when interpreting these findings. The retrospective, single-center design and small sample size limit generalizability and increase the risk of overfitting, even though bootstrap optimism correction was applied. Although the primary mortality model had a reasonable event-to-predictor ratio, the number of non-events was small, and calibration estimates therefore remain uncertain. Calibration slope and calibration-in-the-large/intercept are reported as descriptive point estimates, and interval estimates for calibration were not reported because they were unstable and non-informative in this small cohort. In addition, because predictor prioritization and final model fitting were performed in the same cohort, bootstrap validation of the fixed final model may not fully capture uncertainty introduced by the preceding variable-selection process.

The exploratory secondary endpoints were more strongly constrained by endpoint structure, event distribution, and clinical decision processes. The hemodialysis endpoint was based on few events and was susceptible to competing-risk bias because patients who died before dialysis initiation were classified as “no dialysis” according to the recorded endpoint definition. The *post hoc* Fine-Gray sensitivity analysis partly addressed this issue but remained exploratory and underpowered. ICU admission was imbalanced and may partly reflect center-specific admission pathways and resource use rather than a purely future clinical event. These considerations support the interpretation of the hemodialysis and ICU models as exploratory only.

Missing predictor values were handled using prespecified single imputation. Although missingness in the final predictors was low, single imputation may introduce bias if missingness is informative. In this retrospective hospitalized cohort, missingness may reflect clinical testing patterns and illness severity rather than occurring completely at random. Complete missingness and imputation details are provided in **Supplementary Table 1**.

Although all cases underwent structured chart review by two independent nephrology reviewers using contemporary ADQI/ICA criteria, retrospective adjudication of HRS-AKI remains challenging because AKI phenotypes in advanced cirrhosis may overlap. Mixed HRS-AKI/acute tubular injury phenotypes cannot be excluded with certainty in all cases, particularly when urinary indices or imaging were obtained as part of routine care rather than according to a prospective protocol. However, available urinary findings, imaging reports, treatment documentation, and clinical assessments were reviewed to exclude cases in which an alternative primary cause of AKI was more likely.

Laboratory assay and unit differences, particularly for CRP, may affect transportability and calibration across sites. We did not systematically collect ACLF grade, transplant-listing status, or formal treatment-limitation variables, and no patient underwent liver transplantation during the 90-day observation period. Treatment exposures, including albumin and vasoconstrictor therapy, were reviewed as part of HRS-AKI case ascertainment but were not incorporated as predictors because the model was intentionally restricted to admission laboratory variables. Similarly, treatment response and evolving in-hospital variables, such as early creatinine kinetics or response to vasoconstrictor and albumin therapy, may add prognostic information but would compromise the admission-only, leakage-resistant design objective. External validation in independent cohorts is therefore required before clinical implementation or threshold-based decision-making.

### Implications and next steps

Taken together, these results support the clinical plausibility of a leakage-resistant admission-based risk-stratification approach using four routine laboratory variables. The model is not intended to replace MELD-family scores or to guide clinical decisions in its current form. Rather, it provides a preliminary internally validated framework that may complement future HRS-AKI prognostication research after external validation.

The most important next step is validation in independent, ideally multicenter, cohorts to assess transportability of discrimination and, critically, calibration. Validation should include recalibration when necessary before any threshold-based implementation. Future work should also explore endpoint-specific approaches, including competing-risk or time-to-event modeling for dialysis and ICU outcomes, which are heavily influenced by practice patterns and competing events.

## Conclusion

We developed and internally validated a parsimonious admission-based four-laboratory risk-stratification model for 90-day mortality in hospitalized patients with HRS-AKI. The model demonstrated moderate internally validated discrimination and clinically plausible predictor structure, supporting further evaluation as an early prognostic risk-stratification framework. Given the single-center derivation design, residual optimism, and uncertain calibration, external validation and potential recalibration are required before clinical implementation or threshold-based use. Exploratory secondary endpoint models for in-hospital hemodialysis and ICU admission showed limited performance and should not be used for clinical decision-making in their current form.

## Data Availability

The datasets presented in this article are not readily available due to data protection regulations, patient confidentiality, and institutional restrictions. Requests to access the datasets should be directed to the corresponding author and will be considered on reasonable request, subject to institutional approvals and applicable data protection regulations.

## References

[1] Ginès P Solà E Angeli P Wong F Nadim MK Kamath PS . Hepatorenal syndrome. Nat Rev Dis Primers. (2018) 4:23. doi: 10.1038/s41572-018-0022-7 30213943

[2] Angeli P Garcia-Tsao G Nadim MK Parikh CR . News in pathophysiology, definition and classification of hepatorenal syndrome: A step beyond the International Club of Ascites (ICA) consensus document. J Hepatol. (2019) 71:811–22. doi: 10.1016/j.jhep.2019.07.002 31302175

[3] Biggins SW Angeli P Garcia-Tsao G Ginès P Ling SC Nadim MK . Diagnosis, evaluation, and management of ascites, spontaneous bacterial peritonitis and hepatorenal syndrome: 2021 practice guidance by the American Association for the Study of Liver Diseases. Hepatology. (2021) 74:1014–48. doi: 10.1002/hep.31884 33942342

[4] Nadim MK Kellum JA Forni L Francoz C Asrani SK Ostermann M . Acute kidney injury in patients with cirrhosis: Acute Disease Quality Initiative (ADQI) and International Club of Ascites (ICA) joint multidisciplinary consensus meeting. J Hepatol. (2024) 81:163–83. doi: 10.1016/j.jhep.2024.03.031 38527522 PMC11193657

[5] Pugh RN Murray-Lyon IM Dawson JL Pietroni MC Williams R . Transection of the oesophagus for bleeding oesophageal varices. Br J Surg. (1973) 60:646–9. doi: 10.1002/bjs.1800600817 4541913

[6] Kamath PS Wiesner RH Malinchoc M Kremers W Therneau TM Kosberg CL . A model to predict survival in patients with end-stage liver disease. Hepatology. (2001) 33:464–70. doi: 10.1016/s0016-5085(01)80377-2 11172350

[7] Jalan R Saliba F Pavesi M Amoros A Moreau R Ginès P . Development and validation of a prognostic score to predict mortality in patients with acute-on-chronic liver failure. J Hepatol. (2014) 61:1038–47. doi: 10.1016/j.jhep.2014.06.012 24950482

[8] Chiavegatto Filho A Batista AFM Dos Santos HG . Data leakage in health outcomes prediction with machine learning. Comment on "Prediction of incident hypertension within the next year: Prospective study using statewide electronic health records and machine learning. J Med Internet Res. (2021) 23:e10969. doi: 10.2196/10969 33570496 PMC7880048

[9] Steyerberg EW Harrell FE Jr. Borsboom GJ Eijkemans MJ Vergouwe Y Habbema JD . Internal validation of predictive models: Efficiency of some procedures for logistic regression analysis. J Clin Epidemiol. (2001) 54:774–81. doi: 10.1016/s0895-4356(01)00341-9 11470385

[10] Collins GS Dhiman P Ma J Schlussel MM Archer L Van Calster B . Evaluation of clinical prediction models (part 1): From development to external validation. BMJ. (2024) 384:e074819. doi: 10.1136/bmj-2023-074819 38191193 PMC10772854

[11] Collins GS Reitsma JB Altman DG Moons KG . Transparent reporting of a multivariable prediction model for individual prognosis or diagnosis (TRIPOD): The TRIPOD statement. BMJ. (2015) 350:g7594. doi: 10.1016/j.jclinepi.2014.11.010 25569120

[12] Biggins SW Kim WR Terrault NA Saab S Balan V Schiano T . Evidence-based incorporation of serum sodium concentration into MELD. Gastroenterology. (2006) 130:1652–60. doi: 10.1053/j.gastro.2006.02.010 16697729

[13] Kim WR Mannalithara A Heimbach JK Kamath PS Asrani SK Biggins SW . MELD 3.0: The Model for End-Stage Liver Disease updated for the modern era. Gastroenterology. (2021) 161:1887–1895.e4. doi: 10.1053/j.gastro.2021.08.050 34481845 PMC8608337

[14] Wolff RF Moons KGM Riley RD Whiting PF Westwood M Collins GS . PROBAST: A tool to assess the risk of bias and applicability of prediction model studies. Ann Intern Med. (2019) 170:51–8. doi: 10.7326/m18-1376 30596875

[15] Cervoni JP Thévenot T Weil D Muel E Barbot O Sheppard F . C-reactive protein predicts short-term mortality in patients with cirrhosis. J Hepatol. (2012) 56:1299–304. doi: 10.1016/j.jhep.2011.12.030 22314431

[16] Moreau R Jalan R Gines P Pavesi M Angeli P Cordoba J . Acute-on-chronic liver failure is a distinct syndrome that develops in patients with acute decompensation of cirrhosis. Gastroenterology. (2013) 144:1426–37:37.e1-9. doi: 10.1053/j.gastro.2013.02.042 23474284

[17] Arroyo V Moreau R Jalan R Ginès P . Acute-on-chronic liver failure: A new syndrome that will re-classify cirrhosis. J Hepatol. (2015) 62:S131–43. doi: 10.1016/j.jhep.2014.11.045 25920082

[18] Riley RD Ensor J Snell KIE Harrell FE Jr. Martin GP Reitsma JB . Calculating the sample size required for developing a clinical prediction model. BMJ. (2020) 368:m441. doi: 10.1136/bmj.m441 32188600

