# Supplementary material for: A four-parameter laboratory risk stratification model at admission for 90-day mortality in hepatorenal syndrome-acute kidney injury: a single-center derivation and internal validation study
Source: Front Nephrol. 2026 Jul 17;6:1796660. doi: 10.3389/fneph.2026.1796660 (PMC13423851; doi:10.3389/fneph.2026.1796660)
Supplement: Supplementary file 1 [file DataSheet1.docx]

**SUPPLEMENTARY MATERIAL**

**Table S1.** Missingness matrix and imputation summary for candidate admission predictors

| Variable | Category | Type | Unit | n available | n missing | Missing, % | Imputation method | Imputation value |
| --- | --- | --- | --- | --- | --- | --- | --- | --- |
| Male sex | Demographics | Binary | - | 77 | 0 | 0.0 | Not imputed | - |
| Age | Demographics | Continuous | years | 77 | 0 | 0.0 | Not imputed | - |
| Arterial hypertension | Comorbidity / clinical feature | Binary | - | 77 | 0 | 0.0 | Not imputed | - |
| Hepatic encephalopathy | Comorbidity / clinical feature | Binary | - | 77 | 0 | 0.0 | Not imputed | - |
| Diabetes mellitus | Comorbidity / clinical feature | Binary | - | 77 | 0 | 0.0 | Not imputed | - |
| Smoking | Comorbidity / clinical feature | Binary | - | 77 | 0 | 0.0 | Not imputed | - |
| Systolic blood pressure | Admission vital sign | Continuous | mmHg | 69 | 8 | 10.4 | Median | 117 |
| Heart rate | Admission vital sign | Continuous | /min | 68 | 9 | 11.7 | Median | 88.5 |
| Creatinine | Admission laboratory | Continuous | mg/dL | 77 | 0 | 0.0 | Not imputed | - |
| INR | Admission laboratory | Continuous | unitless | 74 | 3 | 3.9 | Median | 1.54 |
| Leukocytes | Admission laboratory | Continuous | 10⁹/L | 76 | 1 | 1.3 | Median | 11.0 |
| Hemoglobin | Admission laboratory | Continuous | g/dL | 76 | 1 | 1.3 | Median | 10.0 |
| Platelets | Admission laboratory | Continuous | 10⁹/L | 76 | 1 | 1.3 | Median | 96.0 |
| Calcium | Admission laboratory | Continuous | mmol/L | 68 | 9 | 11.7 | Median | 1.10 |
| Lactate | Admission laboratory | Continuous | mg/dL | 68 | 9 | 11.7 | Median | 28.05 |
| Urea | Admission laboratory | Continuous | mg/dL | 67 | 10 | 13.0 | Median | 84.0 |
| Sodium | Admission laboratory | Continuous | mmol/L | 77 | 0 | 0.0 | Not imputed | - |
| Potassium | Admission laboratory | Continuous | mmol/L | 77 | 0 | 0.0 | Not imputed | - |
| Total bilirubin | Admission laboratory | Continuous | mg/dL | 75 | 2 | 2.6 | Median | 4.5 |
| Albumin | Admission laboratory | Continuous | g/dL | 76 | 1 | 1.3 | Median | 2.67 |
| AST | Admission laboratory | Continuous | U/L | 77 | 0 | 0.0 | Not imputed | - |
| ALT | Admission laboratory | Continuous | U/L | 77 | 0 | 0.0 | Not imputed | - |
| C-reactive protein | Admission laboratory | Continuous | mg/dL | 77 | 0 | 0.0 | Not imputed | - |

| – |
| --- |

Missingness is reported for all candidate admission predictors considered during model development. Continuous variables with missing values were imputed using the cohort median; binary variables would have been imputed using the mode if missingness had occurred. Outcomes were not imputed. For the final closed-form four-laboratory models, prespecified single-imputation values were INR 1.54, albumin 2.67 g/dL, and total bilirubin 4.5 mg/dL; C-reactive protein had no missing values and was therefore not imputed. Units are shown as used for model development. Abbreviations: ALT, alanine aminotransferase; AST, aspartate aminotransferase; INR, international normalized ratio.

**Table S2. Patient flow and exclusion reasons**

| Step | n |
| --- | --- |
| ICD-10-coded hepatorenal syndrome cases identified between January 2019 and September 2024 | 174 |
| Excluded before detailed chart review | 32 |
| Duplicate or repeated episodes | 30 |
| Records without hepatorenal syndrome after initial screening | 2 |
| Unique patient records undergoing detailed chart review | 142 |
| Excluded after detailed chart review | 65 |
| No AKI within 7 days after admission | 48 |
| No confirmed cirrhosis with ascites | 16 |
| Structural kidney disease as the primary explanation for kidney dysfunction | 1 |
| Final analytic cohort with chart-review-confirmed HRS-AKI | 77 |

Patients were initially identified through ICD-10 coding for hepatorenal syndrome and subsequently underwent detailed chart review according to contemporary ADQI/ICA criteria. The final analytic cohort comprised 77 patients with chart-review-confirmed HRS-AKI. All endpoint-specific analyses were performed in the full analytic cohort (n=77). Abbreviations: ADQI, Acute Disease Quality Initiative; AKI, acute kidney injury; HRS-AKI, hepatorenal syndrome–acute kidney injury; ICA, International Club of Ascites.

**Supplementary Figure S1. Random-forest variable-importance rankings for admission candidate predictors.**

Random-forest variable-importance rankings are shown for the three study endpoints: S1A, 90-day all-cause mortality; S1B, in-hospital hemodialysis; and S1C, ICU admission. Variable importance is expressed as mean decrease in impurity. Random forests were used only for exploratory prioritization of admission candidate variables and not as final prediction models. Final logistic regression models used a prespecified parsimonious four-laboratory predictor set selected based on clinical plausibility, routine availability, interpretability, and non-redundant representation of hepatic synthetic dysfunction, cholestatic/hepatocellular disease burden, and systemic inflammation. Importance rankings for secondary endpoints should be interpreted cautiously because of limited hemodialysis event counts and ICU class imbalance.

**Supplementary Figure S2. Descriptive calibration plots for exploratory secondary endpoint models.**Descriptive calibration plots are shown for the exploratory secondary endpoint models: S2A, in-hospital hemodialysis, and S2B, ICU admission. Patients were grouped by predicted risk for graphical display; points represent grouped mean predicted probabilities and corresponding observed event proportions. The dashed diagonal line indicates perfect calibration. Predicted probabilities were generated from endpoint-specific closed-form logistic regression equations using INR, albumin, ln(total bilirubin), and ln(C-reactive protein). These plots are intended for descriptive visualization only and should not be interpreted as formal evidence of calibration. Interpretation is limited by the small number of hemodialysis events, the competing-risk structure introduced by death before dialysis, the small cohort size, and ICU class imbalance.
